# Pharmacokinetics of Psilocybin: A Systematic Review

**DOI:** 10.3390/pharmaceutics17040411

**Published:** 2025-03-25

**Authors:** Shakila Meshkat, Huda Al-Shamali, Argyrios Perivolaris, Trusha Tullu, Richard J. Zeifman, Yanbo Zhang, Lisa Burback, Olga Winkler, Andrew Greenshaw, Muhammad Ishrat Husain, Amy C. Reichelt, Eric Vermetten, Manish K. Jha, Rakesh Jetly, Raimar Loebenberg, Venkat Bhat

**Affiliations:** 1 Interventional Psychiatry Program, St. Michael’s Hospital, Toronto, ON M5B 1W8, Canada; shakila.meshkat@unityhealth.to (S.M.); argyrios.perivolaris@unityhealth.to (A.P.); trusha.tullu@mail.utoronto.ca (T.T.); 2 NYU Center for Psychedelic Medicine, NYU Grossman School of Medicine, New York, NY 10016, USA; richard.zeifman@nyulangone.org; 3 Center for Psychedelic Research, Imperial College London, London SW7 2AZ, UK; 4 Department of Psychiatry, University of Alberta, Edmonton, AB T6G 2R3, Canada; 5 Neuroscience and Mental Health Institute (NMHI), University of Alberta, Edmonton, AB T6G 2R3, Canada; 6 Department of Psychiatry, University of Toronto, Toronto, ON M5S 1A1, Canada; ishrat.husain@camh.ca; 7 Centre for Addiction and Mental Health, Campbell Family Mental Health Research Institute, Toronto, ON M6J 1H4, Canada; 8 Department of Physiology and Pharmacology, Western University, London, ON N6A 3K7, Canada; 9 Adelaide Medical School, University of Adelaide, Adelaide, SA 5005, Australia; 10 Department of Psychiatry, Leiden University Medical Center, 2333 ZG Leiden, The Netherlands; 11 Department of Psychiatry, O’Donnell Brain Institute, UT Southwestern Medical Center, Dallas, TX 75390, USA; 12 Institute of Mental Health Research, University of Ottawa, Royal Ottawa Hospital, Ontario, ON K1Z 7K4, Canada; rakesh@drjetly.com; 13 Faculty of Pharmacy & Pharmaceutical Sciences, University of Alberta, Edmonton, AB T6G 2R3, Canada

**Keywords:** psilocybin, pharmacokinetics, metabolism, CYP450

## Abstract

**Background:** Psilocybin has shown promise in therapeutic applications for mental disorders. Understanding the pharmacokinetics of psilocybin and its active metabolite psilocin is crucial for optimizing its clinical use and minimizing adverse effects. **Methods:** This systematic review involved a comprehensive search across MEDLINE, APA PsycINFO, and Embase databases, from inception to December 2024, identifying original studies that investigated the pharmacokinetics of psilocybin. **Results:** Fourteen studies met the inclusion criteria: eight laboratory-based and six clinical studies. Laboratory studies used animal models or in vitro systems, while clinical studies included 112 healthy human participants. Psilocybin is rapidly dephosphorylated to psilocin, which is absorbed with Tmax values ranging from 1.8 to 4 h following oral administration. Cmax varied dose-dependently, from 8.2 ± 2.8 ng/mL (plasma) to 871 ng/mL (urine). One study reported psilocin bioavailability at 52.7 ± 20%. The volume of distribution was extensive, ranging from 277 ± 92 L to 1016 L, suggesting significant tissue distribution. Psilocin metabolism is primarily mediated by CYP2D6 and CYP3A4, with secondary contributions from monoamine oxidase A. It undergoes further hepatic biotransformation into 4-hydroxyindole-3-acetic acid and 4-hydroxytryptophol. Elimination half-life varied across studies, ranging from 1.5 to 4 h. **Conclusions:** Psilocybin pharmacokinetics demonstrate significant variability based on dosage, route, and species. CYP enzymes play a critical role in its metabolism, highlighting the potential for drug–drug interactions. These findings underscore the importance of further research to elucidate psilocybin’s pharmacokinetic profile, which is assessed in vivo by its active metabolite psilocin.

## 1. Introduction

Psilocybin (4-phosphoryloxy-N, N-dimethyltryptamine), a serotonergic psychedelic belonging to the 5-hydroxytryptaminergic class, was first isolated from *Psilocybe* mushrooms by Albert Hofmann in 1957 and subsequently synthesized by him in 1958 [1]. Initially utilized in psychiatric research during the early to mid-1960s, the clinical use of psilocybin as a treatment for mental health conditions was curtailed following its classification as a Schedule 1 substance in the United States in 1970 [2,3]. Research into psilocybin’s potential therapeutic effects resumed in the mid-1990s, with the compound now considered as the preferred agent for clinical studies on serotonergic psychedelics due to its shorter duration of action of 4–6 h compared to similar classical psychedelic substances [4,5,6,7]. Current research on psilocybin emphasizes its administration in controlled, supportive settings to achieve therapeutic benefits. These studies aim to utilize psychospiritual experiences induced by psilocybin, channeling them into durable therapeutic interventions for individuals with mental health conditions [8,9].

Pharmacokinetics refers to the study of the processes underlying the absorption, distribution, metabolism, and excretion of a drug within the body over time [10]. Understanding pharmacokinetics is essential for the safe and effective use of any drug, as it provides critical information about how the drug is processed within the body to confer its therapeutic availability on target organ systems [11]. This knowledge helps determine appropriate dosing regimens, predict therapeutic efficacy and duration, identify potential side effects, generate contraindications, and understand individual variability in drug response. Additionally, pharmacokinetics plays a vital role in drug development, informing the design of clinical trials and ensuring that medications are used safely and efficiently in clinical practice [11]. Psilocybin is a prodrug that undergoes rapid metabolism in the body to its active form, psilocin (N,N-dimethyl-4-hydroxytryptamine), mostly through gut and hepatic dephosphorylation [12]. The pharmacokinetics of psilocybin and psilocin are influenced by various factors, including the rate of absorption, systemic distribution, and elimination via urine and feces [13]. The role of cytochrome P450 (CYP) enzymes, specifically the CYP 450 family, is critical in the metabolism of psilocin [13]. These enzymes, which are involved in the oxidative metabolism of a wide range of substances, may contribute to the interindividual variability observed in psilocybin’s pharmacokinetic profile [14]. Understanding the interaction between psilocybin, psilocin, and the CYP 450 enzymes is essential for predicting potential drug–drug interactions, optimizing dosing strategies, and minimizing adverse effects in clinical settings.

Research into the pharmacokinetic properties of psilocybin and its interactions with CYP 450 enzymes has been limited. As psilocybin continues to gain attention in clinical and therapeutic settings, a thorough understanding of these interactions becomes increasingly important, particularly for individuals on polypharmacy regimens, including other serotonergic-modulating medications such as selective serotonin reuptake inhibitors (SSRIs), tricyclic antidepressants (TCAs), and monoamine oxidase inhibitors (MAOIs). This systematic review aims to synthesize the available evidence on the pharmacokinetics of psilocybin in humans, with a particular focus on the role of CYP 450 enzymes in its metabolism, and to explore the implications for its clinical use.

## 2. Methods

This systematic review followed Preferred Reporting Items for Systematic Reviews and Meta-Analysis (PRISMA) guidelines [15], and the protocol was registered with PROSPERO (CRD42025633390).

### 2.1. Search Strategy

A comprehensive search was completed on three databases: MEDLINE (Ovid Interface, 1946–2024), APA PsycINFO (Ovid Interface, 1974–2024), and Embase (Ovid Interface, 1974–2024) through OVID from inception to December 2024. The following keywords were used: psilocybin psilocyb* or psilocib* or psilocin* or silocyb* or shrooms or magic mushrooms or mushies or psilocybin-assisted therapy AND Pharmacokinetics or Metabolism or Biotransformation or cytochromes or Enzymes or metaboli* or pharmacokinetic* or biotransformation or Cytochrome P450 or CYP450 or CYP enzymes or CYP2D6 or CYP1A2 or CYP3A4 or drug–drug interaction or therapeutic* or safe* or drug interaction* or side effects. The complete search strategy is provided in Supplementary Materials (Table S1).

### 2.2. Eligibility Criteria

The eligibility criteria were as follows: Only original research studies were included, encompassing in vitro, in vivo, clinical, and pharmacokinetic investigations that examined the pharmacokinetics of psilocybin. Studies involving human participants or animal models in which psilocybin was administered were eligible. The intervention specifically needed to address psilocybin’s pharmacokinetics or its interactions with CYP enzymes. Eligible studies reported pharmacokinetic outcomes related to psilocybin’s metabolism, the role of CYP enzymes, or inhibitory effects. Additionally, only studies published in English were considered. Studies were excluded if they focused on non-mammalian models (e.g., insects or plants) that were not directly translatable to human metabolism, if they lacked a pharmacokinetic focus (e.g., studies addressing only clinical efficacy), or if they were narrative reviews, systematic reviews, meta-analyses, conference abstracts, editorials, or opinion pieces.

Our focus on CYP enzymes derives from their central role in psilocybin metabolism and their potential impact on interindividual variability and drug–drug interactions. While there are other metabolic pathways, CYP enzymes are primarily responsible for its biotransformation. CYP polymorphisms are well documented in the literature, leading to significant differences in psilocin metabolism across individuals, which may influence both therapeutic outcomes and adverse effects. Given that CYP enzymes directly influence psilocin’s pharmacokinetic profile, understanding their role is essential for optimizing dosing strategies, minimizing variability in treatment response, and predicting possible interactions with other medications.

### 2.3. Data Screening and Extraction

The screening process was conducted using Covidence (https://www.covidence.org; Melbourne, VIC, Australia, accessed on 22 December 2024). All articles were imported into the platform, where duplicates were automatically identified and removed. Title and abstract screening, followed by full-text review, was performed independently by two reviewers (SM and HAS). Any conflicts were resolved through discussion or, when necessary, by consulting a third reviewer (TT).

The full-texts of eligible studies were reviewed by two independent reviewers (SM and HAS), and the following data were extracted: author names; year of publication; country of the study; study design; participant or animal information; details of psilocybin administration (dosage, route of administration, and frequency); pharmacokinetic parameters (absorption, distribution, metabolism, and excretion); pharmacodynamic effects; adverse events; number and reasons for dropouts; main findings; limitations.

### 2.4. Risk of Bias Assessment

The risk of bias assessment was conducted using the Joanna Briggs Institute (JBI) [16] appraisal tools and the ToxRTool (Toxicological Data Reliability Assessment Tool) [17], with the appropriate tool selected based on the study design, including randomized controlled trials (RCTs), analytical cross-sectional studies, case reports, case series, cohort studies, or in vivo research. Each JBI question in the assessment was rated as “yes”, “no”, “unclear”, or “not applicable”. Unlike tools that generate a summative quality score, the JBI tools emphasize an evaluative approach, where the inclusion decisions were based on the assessor’s judgment regarding each study’s contribution to meaningful insights into psilocybin’s pharmacokinetics. The ToxRTool consists of two different questionnaires, one for in vivo and one for in vitro data. The ToxRTool had each question rated as either “0” or “1”, with the value of “1” representing meeting a criterion. The risk of bias assessment was initially performed independently by one reviewer, after which all reviewers (SM and AP) had the opportunity to review and confirm the results.

### 2.5. Results Synthesis

A narrative analysis was performed in accordance with the SWiM (Synthesis Without Meta-Analysis) [18] methodology, focusing on aggregated data. Studies were categorized into groups, standardized outcome measures were analyzed, and sources of heterogeneity were examined. The extracted data and aggregated results are summarized in evidence tables, with the limitations of the synthesis discussed in detail.

## 3. Results

**Figure 1:**
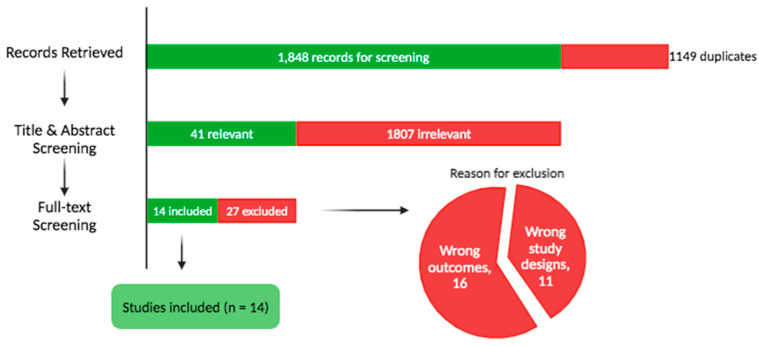
Study flow diagram.

The database search yielded 2997 articles, from which 1149 duplicates were automatically removed, leaving 1848 articles for screening. Following title and abstract review, 41 studies proceeded to full-text assessment, and 14 were ultimately included in this review (Figure 1, Table 1) [13,19,20,21,22,23,24,25,26,27,28,29,30,31].

### 3.1. Risk of Bias Assessment Results

Included articles were evaluated using the JBI appraisal tools and the ToxRTool. In total, six articles were assessed using one of the JBI tools; three articles were assessed using the appraisal tool for RCTs, and three with the quasi-experimental tool. Eight studies were evaluated using the ToxRTool; three articles were assessed using the in vivo questionnaire, two were assessed using the in vitro questionnaire, and three used both in vivo and in vitro. All included studies were deemed to be of sufficient quality to be included in this review. The results of the quality assessment are presented in Supplementary Materials (Table S2).

### 3.2. Study and Sample Characteristics

**Figure 2:**
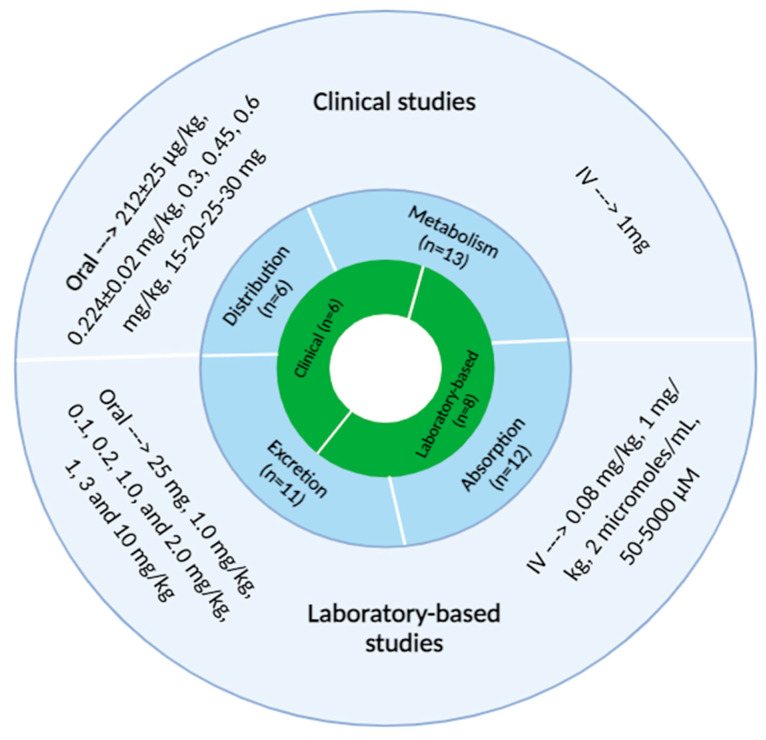
Summary of included study characteristics. This figure categorizes the reviewed studies based on pharmacokinetic parameters (absorption, distribution, metabolism, and excretion) and their study type (clinical or laboratory based). The outer ring illustrates the range of dosing regimens investigated. Abbreviations: IV, intravenous.

Of the fourteen included articles, eight were laboratory-based studies [20,21,25,26,28,29,30,31] and six were clinical studies (Figure 2) [13,19,22,23,24,27]. The laboratory studies involved rats (n = 3 studies) [20,25,30], pigs (n = 1 study) [21], mice (n = 2 studies) [29,31], plasma-based experiments (n = 1 study) [26], or investigations focused on UDP-glucuronosyltransferase (UGT) activity (n = 1 study) [28]. The clinical studies, all conducted in healthy human participants, included a total of 112 individuals. Collectively, these studies explored various aspects of psilocybin’s pharmacokinetics: eleven focused on absorption [13,19,20,21,22,23,24,26,27,29,31], six examined distribution [13,20,21,22,24,27], thirteen investigated metabolism [13,19,20,21,22,23,24,25,27,28,29,30,31], and eleven addressed excretion [13,19,20,21,22,23,24,26,27,29,31].

The studies explored various psilocybin dosages, routes of administration, and frequencies. Dosages ranged from as low as 0.08 mg/kg administered intravenously in pigs to as high as 10 mg/kg administered orally in mice. In humans, oral dosing was the primary route, with fixed doses such as 15 mg, 25 mg, and 30 mg, or weight-based dosages like 0.224 ± 0.02 mg/kg and 0.3–0.6 mg/kg. Frequency was typically limited to single doses, with some studies incorporating repeated measures or crossover designs to evaluate pharmacokinetics under different conditions (Figure 2). All studies administered synthetic psilocybin, except for two animal studies, one explicitly using naturally derived psilocybin (G. spectabilis) [20] and the other not specifying whether the psilocybin was synthetic or natural [21].

### 3.3. Pharmacokinetics

#### 3.3.1. Absorption

The eleven studies investigated the absorption of psilocybin in clinical (n = 6) [13,19,22,23,24,27] and laboratory studies (n = 5) [20,21,26,29,31], reporting data on time to maximum concentration (T_max_), maximum plasma concentrations (C_max_), and bioavailability.

**Figure 3:**
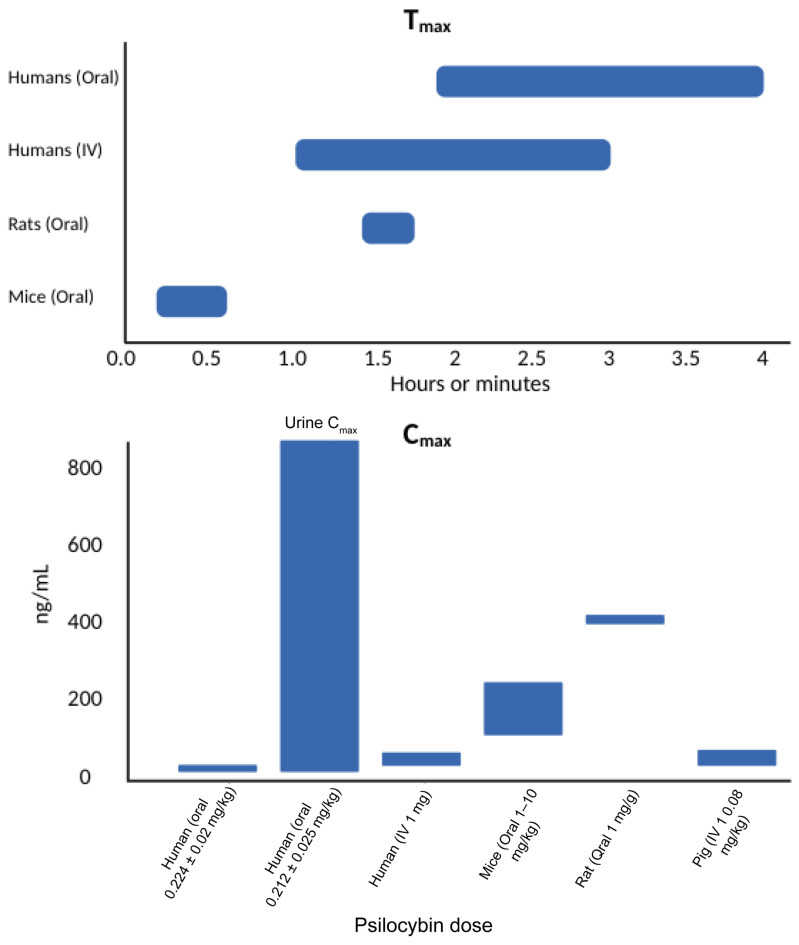
T_max_ and C_max_ of psilocybin across included studies.

T_max_ for psilocin, the metabolite of psilocybin, ranged widely depending on study conditions. The majority of the studies administered psilocybin orally, which resulted in T_max_ values typically around 2 h, with values ranging from 1.8 to 4 h in humans, 1.5 h in rats, and 15–30 min in mice. For intravenous (IV) administration, T_max_ was significantly shorter at 1.9 ± 1.0 min (0.03 h) as assessed by one study in a healthy human sample (Figure 3).

C_max_ for psilocin varied widely across studies. In humans, oral administration resulted in plasma C_max_ mean values ranging from 8.2 ± 2.8 ng/mL (psilocybin dose = 0.224 ± 0.02 mg/kg) [22] to 97 ± 33 ng/mL (psilocybin dose = 25 mg) [19]. Additionally, one study reported a urine C_max_ of 871 ng/mL following a psilocybin dose of 0.212 ± 0.025 mg/kg [23]. Some studies suggest that C_max_ is dose dependent. A pre–post study involving 12 healthy participants observed that C_max_ increased with escalating oral doses of psilocybin: 16 ng/mL at 0.3 mg/kg, 26 ng/mL at 0.45 mg/kg, and 37.6 ng/mL at 0.6 mg/kg [13]. Another study, a randomized controlled crossover trial with 28 participants, confirmed the dose dependency, reporting C_max_ values of 13 ng/mL for a 15 mg dose and 25 ng/mL for a 30 mg dose, both administered orally [24]. All human studies reporting C_max_ used oral administration, except one study, which administered psilocybin intravenously (1 mg) and found a C_max_ of 12.9 ± 5.6 ng/mL [22].

There were four animal studies that examined absorption: two on mice, one on rats, and one on pigs. The mouse studies, all using oral administration, investigated doses of 1 mg/kg (C_max_ = 52.9 ng/mL), 3 mg/kg (C_max_ = 198 ± 28 ng/mL), and 10 mg/kg (C_max_ = 243 ng/mL) [29,31]. The rat study, also using oral administration (1 mL/g), reported a C_max_ of 430 ng/mL [20]. In the pig study, an intravenous (IV) dose of 0.08 mg/kg resulted in a C_max_ ranging from 12 to 19 ng/mL (Figure 3) [21].

Bioavailability was examined in two studies. In one study, psilocin demonstrated a bioavailability of 52.7 ± 20% when administered orally at a dose of 0.224 ± 0.02 mg/kg [22]. The other study reported high bioavailability, although no specific percentage was provided [29].

#### 3.3.2. Distribution

Six studies investigated the distribution of psilocybin, providing data on the volume of distribution (Vd) [13,20,21,22,24,27]. Reported Vd values varied depending on the population, dose, and route of administration. In humans, Vd for psilocin ranged from 277 ± 92 L for intravenous administration [22] to values as high as 1016 L for oral doses of 30 mg [24]. Another study with oral administration of escalating doses (0.3–0.6 mg/kg) estimated a Vd of 298 L [13]. In animal studies, Vd values were also reported, with a rat study showing 3.2 ± 1.7 mL [20] and a pig study reporting 4.2 mL/cm^3^ [21]. These findings suggest that psilocin exhibits a large Vd, indicating extensive tissue distribution.

#### 3.3.3. Metabolism

**Figure 4:**
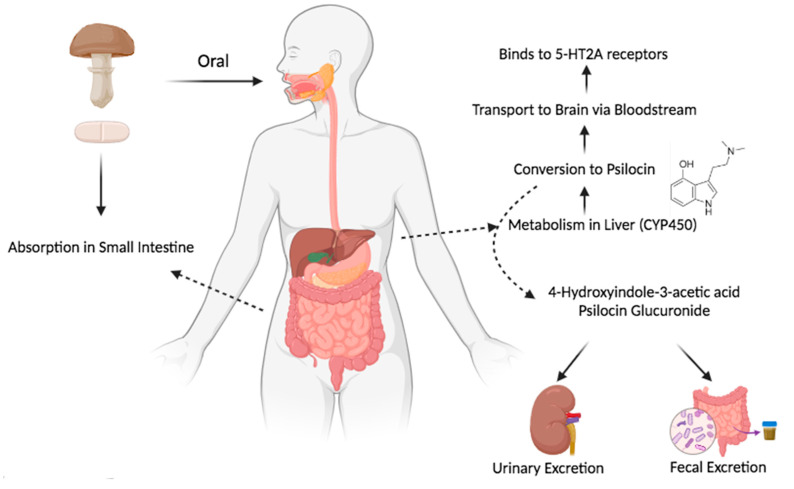
Oral psilocybin metabolism and excretion pathway.

Six studies reported on the enzymes involved in the metabolism of psilocybin [23,25,28,29,30,31], while thirteen studies identified its metabolites [13,19,20,21,22,23,24,25,27,28,29,30,31]. The enzymes mentioned included CYP450 isoforms, with CYP2D6 extensively metabolizing psilocin, and CYP3A4 contributing moderately (approximately 40%). Monoamine oxidase A (MAO-A) catalyzed the formation of secondary metabolites such as 4-hydroxyindole-3-acetic acid (4-HIAA) and 4-hydroxytryptophol (4-HTP). UDP-glucuronosyltransferases (UGTs), particularly UGT1A9 and UGT1A10, were implicated in the glucuronidation of psilocin (Figure 4). The primary metabolite of psilocybin reported across studies is psilocin, which undergoes further transformation into secondary metabolites such as 4-HIAA, 4-HTP, and psilocin-O-glucuronide. Additional minor metabolites include oxidized psilocin and, in some studies with animal models, norpsilocin.

#### 3.3.4. Half-Life and Excretion

Eleven studies investigated the excretion of psilocybin, focusing on half-life (t_1/2_), clearance rates, and routes of excretion [13,19,20,21,22,23,24,26,27,29,31]. The half-life of psilocin varied widely depending on the species and administration route. In humans, oral administration resulted in t_1/2_ values of approximately 2–4.8 h for psilocin and 1.7–2.4 h for its primary metabolite, 4-HIAA [13,19,22,23,24,26,27]. In contrast, intravenous administration led to a much shorter t_1/2_ for psilocin, around 74.1 ± 19.6 min (1.2 h) [22].

Animal studies showed comparable variability. In mice, the oral administration of 3 mg/kg psilocybin resulted in a t_1/2_ of 0.91 ± 0.11 h [31]. Similarly, in rats given 1 mL/g orally, the distribution t_1/2_ was 1.95 ± 0.67 h, while the elimination t_1/2_ was slightly longer at 2.5 ± 1 h [20]. In pigs receiving an intravenous dose of 0.08 mg/kg, the t_1/2_ was notably short, at only 20 min (0.3 h) [21].

Clearance rates varied, with values ranging from 155 L/h (psilocybin = 20 mg) to 263 L/h (psilocybin = 30 mg) following oral administration in humans [13,24,27] and 188 ± 43 L/h for IV dosing [22]. In animal studies, a study on rats reported a clearance rate of 0.132 ± 0.054 L/h following oral administration (1 mL/g) [20]. The primary route of excretion for psilocin was only reported in four studies involving healthy human participants who received psilocybin orally; all studies identified renal excretion as the dominant pathway [13,23,26,27].

## 4. Discussion

In this review on the pharmacokinetics of psilocybin, significant variability was observed across studies, influenced by factors such as dose, species, and route of administration. Psilocybin was found to be rapidly absorbed, with Tmax typically reported around 2 h following oral administration and much shorter with intravenous dosing. Extensive distribution was noted, with a high volume of distribution (up to 1016 L in humans), indicating widespread tissue penetration. Psilocybin was metabolized to its active compound, psilocin, primarily through the actions of CYP2D6 and CYP3A4 enzymes, with further metabolism producing secondary compounds such as 4-HIAA and psilocin-O-glucuronide. Elimination was shown to occur predominantly via renal excretion, with the half-life of psilocin ranging from 2 to 4.8 h following oral administration. These findings highlight the complexity of psilocybin’s pharmacokinetics and provide important insights for its therapeutic use.

IV administration of psilocybin led to a markedly shorter Tmax for psilocin, compared to psilocin Tmax after oral psilocybin administration. This rapid conversion of psilocybin to psilocin, with a Tmax of just 2 min, is due to the direct absorption of psilocybin into the bloodstream, bypassing first-pass hepatic metabolism [32]. In contrast, the oral administration of psilocybin results in delayed psilocin absorption due to the time required for the compound to pass through the gastrointestinal system, in addition to undergoing first-pass metabolism in the liver [32]. If first-pass metabolism was not involved, we would expect IV administration to result in lower psilocin levels and higher psilocybin levels relative to the oral route [33]. However, the rapid conversion of psilocybin to psilocin after IV administration highlights the difference in absorption and metabolism between the two routes. While this rapid absorption may enhance therapeutic efficacy in situations requiring a quick onset of action, the limitations of IV administration—such as its invasive nature, the need for specialized medical infrastructure, and the potential for adverse reactions due to rapid changes in plasma concentration—restrict its practical use in routine therapy [34]. Additionally, the wide range of Cmax values observed across studies underscores variability in psilocybin metabolism, influenced by individual factors like enzymatic activity and study conditions. Dose-dependent increases in Cmax confirm the predictability of psilocybin’s pharmacokinetics, but interindividual and inter-species differences, such as higher plasma concentrations in animal studies, highlight the need for caution when extrapolating these results to humans [13,21,22,24,29,31]. These findings emphasize the potential importance of personalized dosing strategies and the need for further research to optimize psilocybin’s therapeutic use.

The oral administration of psilocybin results in a higher Vd compared to IV administration. This difference is likely influenced by various factors, including metabolism, plasma protein binding, the route of administration, and the drug’s intrinsic properties [13,20,21,22,24,27]. While a high Vd may suggest extensive tissue distribution, it does not necessarily indicate a reduced potential for toxicity. In some cases, drugs with high Vds can be highly toxic due to prolonged retention in tissues and difficulty eliminating them [35,36]. Additionally, the high Vd may indicate extensive distribution into the central nervous system (CNS), which is beneficial for its therapeutic effects, especially in treating psychiatric conditions [37]. However, this extensive distribution could present challenges in monitoring and the slower elimination of the drug, which may require careful management in clinical settings [38]. Additionally, oral administration leads to a longer t1/2 of 2–4.8 h compared to approximately 1.2 h for IV administration. This difference reflects faster systemic clearance rates of 3126 ± 719 mL/min for IV dosing versus more variable oral clearance rates, which range from 60.6 µg/h to 263 L/h [39]. The longer half-life observed with oral dosing may play a role in the sustained therapeutic effects seen in psychiatric treatments, though the exact relationship between pharmacokinetics and clinical duration remains unclear. In contrast, the shorter half-life of IV administration could be advantageous for conditions requiring precise dosing and the rapid cessation of effects, though its clinical implications warrant further investigation [40]. Additionally, cost-effectiveness may be an important consideration, as oral administration is generally more accessible and less resource-intensive compared to IV delivery, which requires medical supervision and specialized equipment. Renal excretion is the primary elimination pathway for psilocin, emphasizing the critical role of kidney function in its clearance and explaining why clinical trials commonly exclude individuals with renal failure to avoid potential accumulation and toxicity. This exclusion highlights the need for caution when considering psilocybin therapy in populations with chronic renal disease [41,42]. Additionally, the variability in clearance rates and half-life across species highlights the complexity of psilocybin’s metabolism and excretion. Future research should address the challenges by optimizing dosing strategies and evaluating safety in diverse patient populations, including those with renal impairment.

Psilocybin is metabolized via multiple enzymatic pathways, starting with alkaline phosphatase to psilocin followed by CYP450, MAO-A, and UGTs, which play a significant role in its pharmacokinetics and potential drug interactions. Psilocybin undergoes rapid metabolism after administration, primarily converting to its active metabolite, psilocin, through enzymatic pathways. Psilocin, the primary mediator of its psychoactive effects, is further metabolized into secondary metabolites such as 4-hydroxyindole-3-acetic acid (4-HIAA) and psilocin-O-glucuronide. The involvement of CYP450 enzymes, particularly CYP2D6 and CYP3A4, highlights the potential for drug–drug interactions, as substances that inhibit or induce these enzymes could significantly alter psilocybin’s pharmacokinetics [43]. This enzymatic metabolism not only underscores the potential for variability in individual responses but also emphasizes the need for caution when psilocybin is used in combination with other medications [43]. The CYP450 enzyme family, responsible for metabolizing a broad range of medications, is particularly relevant for interactions involving psilocybin [44]. Studies have highlighted possible interactions between psilocybin and various drug classes, including anxiolytics, antipsychotics, mood stabilizers, antidepressants, and recreational substances [45]. For example, co-administration with antipsychotics such as chlorpromazine, risperidone, and haloperidol has been shown to attenuate psilocybin-induced effects, such as visual perceptual changes and the sensation of ego dissolution, with some antipsychotics also influencing working memory performance [46,47,48]. Psilocybin primarily exerts its effects through agonism at serotonin 2A receptors (5-HT2A) [49]. Medications with 5-HT2A antagonist properties, such as risperidone, have been shown to partially or completely block psilocybin’s subjective effects [46,47,48]. Additionally, selective serotonin reuptake inhibitors (SSRIs), like escitalopram, which increase extracellular serotonin by inhibiting its reuptake, may attenuate psilocybin’s acute effects and potentially influence treatment-related outcomes, such as reductions in depression symptoms [19,45]. This attenuation occurs due to receptor competition between elevated endogenous serotonin and psilocin at serotonin receptors [19]. Interestingly, despite prior expectations, escitalopram pretreatment did not significantly reduce psilocybin’s core subjective effects but did significantly mitigate some physiological reactions, such as increased blood pressure and pupil dilation [19,50]. Notably, mood stabilizers such as lithium and lamotrigine warrant special consideration, as case reports suggest that their combination with psilocybin or other psychedelics may increase the risk of adverse effects, including seizures and manic episodes [51,52].

An important consideration in the metabolism of psilocybin is the distinction between synthetic, purified psilocybin, and naturally occurring psilocybin derived from fungal sources. Naturally sourced psilocybin contains a complex mixture of other tryptamines, such as baeocystin and norbaeocystin, which may also interact with CYP enzymes. These additional tryptamines could compete for enzymatic processing, potentially altering the pharmacokinetics of psilocybin and its active metabolite, psilocin. In contrast, synthetic psilocybin, being a purified compound, follows a more predictable metabolic pathway without interference from other bioactive constituents. However, some studies suggest that naturally sourced psilocybin may produce more robust symptom improvement [53], indicating that the presence of additional bioactive compounds could enhance therapeutic effects rather than hinder them.

Psilocybin’s potential as a psychiatric treatment is due to its pharmacological effects on the serotonergic system and its ability to affect neural networks implicated in mental health disorders. By modulating 5-HT2A receptor activity, psilocybin alters functional connectivity, particularly within the default mode network (DMN), which plays a key role in self-referential thinking and emotional regulation [54,55]. Decreased DMN activity has been associated with a shift in perspective and increased mental flexibility, which may be beneficial in conditions characterized by rigid thought patterns, such as depression [55,56,57]. Psilocybin has also been shown to promote structural neuroplasticity, enhancing dendritic spine density and synaptic strength in preclinical models [58]. These findings align with clinical studies suggesting that psilocybin’s therapeutic effects may extend well beyond the acute psychedelic experience. Despite its classification as a Schedule 1 substance, interest in psilocybin has increased due to evidence from clinical trials demonstrating its potential benefits and a relatively favorable safety profile when administered in controlled settings [59,60]. Understanding psilocybin’s pharmacokinetics, including its metabolism, is essential for optimizing dosing strategies and identifying potential drug–drug interactions. Future research should aim to refine its clinical use, particularly in patient populations with varying metabolic profiles, to maximize therapeutic benefits while minimizing risks.

This systematic review has several limitations that should be acknowledged. First, the included studies exhibited significant heterogeneity in methodologies, including variability in psilocybin dosages, routes of administration, study designs, and populations (e.g., human versus animal models). This variability limited our ability to perform a meta-analysis and may have influenced the consistency of reported pharmacokinetic outcomes. Second, the small sample sizes in several human studies (totaling only 112 participants across six studies) may reduce the generalizability of findings to broader populations, particularly in clinical settings. Third, only one study examined intravenous administration, and none were conducted in clinical samples, limiting the applicability of findings to therapeutic contexts. Additionally, there was a lack of dose–response studies, and no study comprehensively assessed both pharmacokinetics and pharmacodynamics. Reporting inconsistencies further hindered comparisons across studies, highlighting the need for standardized guidelines. Given these gaps, future research should prioritize larger, well-controlled trials with standardized methodologies and diverse populations. Moreover, adherence to established pharmacodynamic reporting guidelines, such as those recommended by the Food and Drug Administration or the European Medicines Agency for psychedelic drug development, could improve consistency and comparability across studies.

## 5. Conclusions

This systematic review provides a comprehensive synthesis of psilocybin’s pharmacokinetics, highlighting key findings across absorption, distribution, metabolism, and excretion. Oral administration remains the most commonly studied route, with absorption characterized by Tmax values around 2 h and dose-dependent Cmax levels. Distribution data indicate a large volume of distribution for psilocin, supporting extensive tissue uptake. Metabolism primarily involves CYP enzymes, particularly CYP2D6 and CYP3A4, with secondary contributions from UGTs and MAO-A, resulting in both active and inactive metabolites. Excretion studies consistently identify renal clearance as the primary elimination pathway. Despite variability in reported pharmacokinetic parameters due to differences in study design, populations, and dosing regimens, these findings underscore the complexity of psilocybin metabolism and its potential for drug–drug interactions.

## 6. Future Directions

Future research should focus on refining psilocybin’s pharmacokinetic profile across diverse populations, including individuals with varying genetic polymorphisms of CYP enzymes. Further studies are needed to evaluate potential drug–drug interactions, particularly with medications metabolized by CYP2D6 and CYP3A4. Additionally, investigating alternative routes of administration, such as intranasal or sublingual delivery, may optimize bioavailability and therapeutic outcomes. Standardizing study methodologies, including consistent dosing protocols and analytical techniques, will be essential for enhancing comparability across studies. Finally, exploring pharmacokinetic–pharmacodynamic relationships in clinical populations will help optimize dosing strategies for psychiatric and neurological disorders.

## Figures and Tables

**Table 1:** Characteristics of included studies.

Study		Participant Information		Pharmacokinetics				Main Results
Author	Study Design	Participants	Psilocybin	Absorption	Distribution	Metabolism	Excretion	
Becker et al., 2022 [19]	Clinical: RCT Crossover	n = 23 Human (healthy) 48% female Mean age = 34 ± 10 yrs	Synthetic 25 mg, single dose administered twice (14 days apart), oral dose	T_max_ (_h_): Escitalopram: Psilocin Unconjugated = 2 Psilocin Glucuronide = 4 Psilocin Total = 3 4-HIAA = 2 Placebo: Psilocin Unconjugated = 2 Psilocin Glucuronide = 4 Psilocin Total = 3 4-HIAA = 2 C_max_ (ng/mL) in Plasma: Escitalopram: Psilocin Unconjugated = 22 ± 8.5 Psilocin Glucuronide = 82 ± 30 Psilocin Total = 97 ± 33 4-HIAA = 106 ± 37 Placebo: Psilocin Unconjugated = 20 ± 5.4 Psilocin Glucuronide = 82 ± 28 Psilocin Total = 96 ± 28 4-HIAA = 105 ± 30	N/R	Enzyme = N/R Metabolites = psilocin, psilocin glucuronide, or 4-HIAA	t_1/2_ (h): Escitalopram: Psilocin Unconjugated = 2.0 ± 0.5 Psilocin Glucuronide = 5.7 ± 2.4 Psilocin total = 4.8 ± 1.8 4-HIAA = 1.7 ± 0.5 Placebo: Psilocin Unconjugated = 1.8 ± 0.3 Psilocin Glucuronide = 4.7 ± 1.6 Psilocin total = 4.3 ± 1.3 4-HIAA = 1.6 ± 0.3	Escitalopram pretreatment did not significantly affect psilocybin’s positive mood effects but reduced its adverse effects (e.g., anxiety and cardiovascular reactions), without altering psilocin pharmacokinetics.
Brown et al., 2017 [13]	Clinical: pre–post study design	n = 12 Human (healthy) 17% female Mean age = 43 yrs	Synthetic Escalating oral dose of 0.3, 0.45, and 0.6 mg/kg	T_max_ (h): 0.3 mg/kg = 2.03 0.45 mg/kg = 2.03 0.6 mg/kg = 2.05 C_max_ (ng/mL) in Plasma: 0.3 mg/kg = 16 0.45 mg/kg = 26 0.6 mg/kg = 37.6	Vd (L) = 298	Enzyme = N/R Metabolites = psilocin and psilocin glucuronide	t_1/2_ (h) = 3 ± 1.1 Clearance (L/h) = 164 ± 23.2 Route of Elimination = Renal	Oral psilocybin demonstrates linear pharmacokinetics over the dose range of 0.3–0.6 mg/kg, with psilocin having a rapid onset, a half-life of approximately 3 h, and minimal renal excretion, suggesting no need for dose adjustment in mild to moderate renal impairment. A fixed 25 mg oral dose approximates the exposure of 0.3 mg/kg.
Chen et al., 2011 [20]	Laboratory: experimental study	n = 10 Sprague Dawley rats	Natural (from *G. spectabilis*) 1 mL/g, single dose, oral gavage	T_max_ (h): Psilocin = 1.5 ± 0.03 C_max_ (ng/mL) in Plasma: Psilocin = 430 ± 120	Vd (L) = 0.0032 ± 0.0017	Enzyme = N/R Metabolite = psilocin	Distribution t_1/2_ (h) = 1.95 ± 0.67 Elimination t_1/2_ (h) = 2.5 ± 1 Clearance (L/h) = 0.132 ± 0.054	The study developed a UPLC-PDA detector to assess psilocin pharmacokinetics in rat plasma, showing rapid absorption post oral administration of Gymnopilus spectabilis extract.
Donovan et al., 2021 [21]	Laboratory: dose–response experimental study	n = 25 Danish slaughter pigs 100% female Mean age = 9 weeks	Unclear 0.08 mg/kg, single dose, IV	C_max_ (ng/mL) in Plasma = 12–19	Non-displaceable Vd (mL/cm^3^) = 4.2	Enzyme = N/R Metabolite = psilocin	t_1/2_ (h) = 0.3	Psilocybin in pigs can induce transient behavioral changes (i.e., headshaking and scratching), achieves 67% occupancy of cerebral 5-HT2A receptors, results in small changes in PFC gene expression, and modulates immune-related gene expression pathways in pigs.
Hasler et al., 1997 [22]	Clinical: controlled clinical trial	n = 9 Human (healthy) 13% female Mean age = 31 ± 6	Synthetic Six participants, single dose (0.224 ± 0.02 mg/kg), orally; six participants, single IV dose (1 mg)	Oral: T_max_ (h): Psilocin = 1.75 ± 0.62 4-HIAA = 1.88 ± 0.68 C_max_ (ng/mL) in Plasma: Psilocin = 8.2 ± 2.8 4-HIAA = 150 ± 61 Bioavailability (%): Psilocin = 52.7 ± 20 IV: T_max_ (h): Psilocin = 0.0317 (1.9 min) C_max_ (ng/mL): Psilocin = 12.9 ± 5.6	IV: Vd (L) = 277 ± 92	Enzyme = N/R Metabolites = psilocin and 4-HIAA	Oral: t_1/2_ (h) = Psilocin = 2.7 ± 1.06 4-HIAA = 2.4 ± 1.61 IV: t_1/2_ (h) = Psilocin = 1.2 ± 0.33 Clearance (L/h) = 187.56 ± 43.14	The study revealed differences between oral and IV psilocybin administration. IV resulted in a rapid peak of psilocin levels, while oral dosing delayed the peak with a longer half-life. 4HIAA was detected only after oral administration, highlighting first-pass metabolism.
Hasler et al., 2002 [23]	Clinical: controlled clinical trial	n = 8 Human (healthy) 50% female Mean age = 33 ± 6	Synthetic 0.212 ± 0.025 (mg/kg), single oral dose	T_max_ (h): Psilocin Unconjugated = 2–4 C_max_ (ng/mL) in urine: Psilocin Unconjugated = 871	N/R	Enzyme = enzymatic glucuronide Metabolite = psilocin	t_1/2_ (h) = 3.29 ± 0.57 Route of Elimination = Renal	Psilocybin is rapidly metabolized to psilocin. Psilocin undergoes partial glucuronidation, extending its detectability, and 3.4% of the administered psilocybin dose is excreted as unconjugated psilocin within 24 h.
Holze et al., 2022 [24]	Clinical: RCT crossover	n = 28 Human (healthy) 50% female Mean age = 35 ± 9.4 yrs	Synthetic 15 mg or 30 mg, single, oral dose	T_max_ (h): Psilocin Unconjugated 15 mg = 2.3 30 mg = 2.5 C_max_ (ng/mL) in Plasma: Psilocin Unconjugated 15 mg = 13 30 mg = 25	Vd (L): 15 mg = 925 30 mg = 1016	Enzyme = N/R Metabolite = psilocin	t_1/2_ (h): 15 mg = 2.4 30 mg = 2.7 Clearance (L/h): 15 mg = 262 30 mg = 263	Psilocybin produced dose-dependent effects on mood and consciousness comparable to LSD but with a shorter duration of action. While both substances exhibit cardiostimulatory effects, psilocybin increases blood pressure more significantly, whereas LSD has a greater impact on heart rate.
Horita et al., 1961 [25]	Laboratory: experimental study	n = N/R Sprague Dawley rats 100% male	Synthetic 2 micromoles/mL, single addition	N/R	N/R	Enzymes = oxidase enzyme and phosphatase enzyme Metabolite = psilocin	N/R	Psilocybin dephosphorylation was most active in the kidneys of rats and mice and the small intestine mucosa of guinea pigs and rabbits. Oxidase activity peaked in the heart across species and in the kidneys of rats and mice. These findings suggest that psilocybin is rapidly converted to its active form, psilocin, with its effect duration potentially regulated by psilocin oxidation to an o-quinone structure.
Kolaczynska et al., 2021 [26]	Laboratory: experimental study	Plasma from n = 3	Synthetic 25 mg single dose, oral	T_max_ (h): Psilocin = 2.3 ± 0.77 Psilocin Glucuronide = 3.67 ± 1.53 4-HIAA = 2 ± 1 C_max_ (ng/mL) in Plasma: Psilocin = 19.2 ± 4.0 Psilocin Glucuronide = 78.3 ± 7.9 4-HIAA = 137 ± 22	N/R	N/R	t_1/2_ (h): Psilocin = 2.1 ± 0.3 Psilocin Glucuronide = 3.58 ± 1.2 4-HIAA = 2.3 ± 1.05 Route of Elimination = Renal	There was rapid metabolism of psilocybin into its active form, psilocin, with efficient glucuronidation and renal elimination. A reliable LC-MS/MS method for quantifying psilocin and its metabolites was developed, providing valuable insights into psilocybin’s pharmacokinetics and supporting its potential for therapeutic use and future research on efficacy and safety.
Ley et al., 2023 [27]	Clinical: RCT	n = 32 Human (healthy) 50% female Mean age = 29 ± 4 yrs	Synthetic 20 mg (four oral capsules of 5 mg each), single dose	T_max_ (h): Psilocin = 2.1 Psilocin Glucuronide = 4.4 4-HIAA = 1.8 h C_max_ (ng/mL) in Plasma: Psilocin = 17 Psilocin Glucuronide = 70 4-HIAA = 86	Vd (L): Psilocin = 505 Psilocin glucuronide = 190 4-HIAA = 116	Enzyme = N/R Metabolites = psilocin, psilocin glucuronide, and 4-HIAA	t_1/2_ (h): Psilocin = 2.3 Psilocin Glucuronide = 3.2 4-HIAA = 2.1 Clearance Rate (L/h): Psilocin = 155 Psilocin Glucuronide = 41 4-HIAA = 37 Route of Elimination = Renal	No qualitative differences in altered states of consciousness were observed between 500 mg mescaline, 100 µg LSD, and 20 mg psilocybin, though their durations of action differed. The findings support dose optimization for research and psychedelic-assisted therapy.
Manevski et al., 2010 [28]	Laboratory: experimental study	19 UGTs	Synthetic 50–5000 uM, single dose	N/R	N/R	Enzymes = UDP-Glucuronosyltransferases (UGTs; UGT1A10, UGT1A9, UGT1A6, and UGT1A8) Metabolites = psilocin and 4-hydroxyindole	N/R	The study revealed that psilocin undergoes extensive glucuronidation, with UGT1A10 playing a key role in first-pass metabolism in the small intestine and UGT1A9, contributing to liver clearance. Substrate specificity was observed, with psilocin primarily metabolized by UGT1A10 and 4-hydroxyindole by UGT1A6, highlighting the tissue-specific roles of UGTs.
Raithatha et al., 2023 [29]	Laboratory: experimental study	Pharmacokinetic studies: n = 12 mice, (n = 3 per dose level group) Head twitch: N = 6 mice Marble burying: n = 36	Synthetic Pharmacokinetics: 1 mg/kg IV, 1,3, or 10 mg/kg oral gavage Behavioral test: 1 mg/kg oral gavage, single dose	T_max_ = 0.25 h C_max_ (oral; ng/mL) in Plasma: 1 mg/kg = 52.9 10 mg/kg = 243 High Bioavailability	N/R	Enzymes = alkalinephosphatase and nonspecific esterases Metabolite: psilocin	Plasma Psilocin Levels Detectable for up to 24 h	Tailored prodrugs (novel psilocin drugs) may be more effective than psilocybin for treating depression and anxiety without unwanted psychedelic effects.
Rakoczy et al., 2023 [30]	Laboratory: experimental study	Head-twitch response: n = 67, Long Evans rats Forced swim: n = 60 Toxicology: n = 15	Synthetic Head-twitch response: psilocybin dosages tested: 0.1, 0.2, 1.0, and 2.0 mg/kg; intraoral gavage; single dose Forced swim test: psilocybin dosage: 1.0 mg/kg; intragastric infusion; 3 times over 24 h Toxicology: psilocybin dosage: 1.0 mg/kg; oral gavage; single dose	N/R	N/R	Enzymes = alkaline phosphatase, and MAO-A Metabolites: psilocin, 4-hydroxyindole-3-acetaldehyde, and 4-HIAA	N/R	In vitro assays revealed similar dephosphorylation and metabolism rates across compounds. Dephosphorylated baeocystin and norbaeocystin crossed a blood–brain barrier mimetic and activated the 5-HT2A receptor with efficacy comparable to psilocin. Only psilocybin induced head-twitch responses in rats, indicating psychedelic effects, while norbaeocystin improved forced swim test outcomes.
Thomann et al., 2024 [31]	Laboratory: controlled experimental study Clinical: secondary analysis (RCT)	Mouse: n = 10 (5 experimental, 5 control) Adult C57BL/6J mice 100% male Human: N = 5 See Holze et al., 2022 [24]	Synthetic Mouse: 3 mg/kg, single, oral gavage needle dose	Mouse: T_max_(h): Psilocin = 0.30 ± 0.11 Psilocin-O-Glucuronide = 0.35 ± 0.14 4-HIAA = 0.30 ± 0.11 4-HIAA-Glucuronide = 0.45 ± 0.11 C_max_ (ng/mL) in Plasma: Psilocin = 198 ± 28 Psilocin-O-Glucuronide = 521 ± 57 4-HIAA = 84.9 ± 17.7 4-HIAA-Glucuronide = 30.0 ± 6.7	N/R	Enzymes = CYP, MAO-A, and UGT Metabolites = psilocin, psilocin-O-glucuronide, 4-HIAA, 4-HTP, oxidized psilocin metabolite, and norpsilocin	Mouse: t_1/2_ (h): Psilocin = 0.91 ± 0.11 Psilocin-O-Glucuronide = 0.97 ± 0.06 4-HIAA = 0.75 ± 0.11 4-HIAA-Glucuronide = 1.38 ± 0.27	Six psilocin metabolites were identified, confirming in vivo glucuronidation and highlighting interspecies differences, such as 4-HIAA glucuronidation and norpsilocin detection in mice but not humans. MAO-A plays a key role in converting psilocin to 4-HIAA and 4-HTP, while the roles of ALDH and ADH remain unclear. CYP2D6 minimally contributes to psilocin metabolism, producing norpsilocin and an oxidized metabolite, while CYP3A4’s role is uncertain.

Abbreviations: 4-HIAA, 4-hydroxyindole-3-acetic acid; 4-HTP, 4-hydroxytryptophol; 5-HT2A, serotonin 2A; ADH, alcohol dehydrogenase; ALDH, aldehyde dehydrogenase; Cmax, maximum plasma concentrations; CYP, cytochrome P450; H, hour; IV, intravenous; L, liter; LC-MS/MS, high-pressure liquid chromatography with tandem mass spectrometry; LSD, lysergic acid diethylamide; MAO-A, monoamine oxidase A; MAO, monoamine oxidase; Min, minutes; ml, milliliter; n, sample size; N/R, not reported; PFC, prefrontal cortex; RCT, randomized controlled trial; T1/2, half-life; Tmax, time to maximum concentration; UGT, UDP-glucuronosyltransferase; UPLC-PDA, ultra-performance liquid chromatography coupled with a photodiode array; Vd, volume of distribution; Yrs, years.

## Supplementary Materials

The following supporting information can be downloaded at: https://www.mdpi.com/article/10.3390/pharmaceutics17040411/s1. Table S1. Full search strategy. Table S2. Risk of bias assessment results.

## References

[1] Hofmann A., Heim R., Brack A., Kobel H. (1958). Psilocybin, a psychotropic substance from the Mexican mushroom Psilicybe mexicana Heim. Experientia.

[2] Passie T., Seifert J., Schneider U., Emrich H.M. (2002). The pharmacology of psilocybin. Addict. Biol..

[3] Passie T., Metzner R. (2004). A History of the Use of Psilocybin in Psychotherapy.

[4] Carhart-Harris R.L., Goodwin G.M. (2017). The therapeutic potential of psychedelic drugs: Past, present, and future. Neuropsychopharmacology.

[5] Carter O.L., Burr D.C., Pettigrew J.D., Wallis G.M., Hasler F., Vollenweider F.X. (2005). Using psilocybin to investigate the relationship between attention, working memory, and the serotonin 1A and 2A receptors. J. Cogn. Neurosci..

[6] Goldberg S.B., Pace B.T., Nicholas C.R., Raison C.L., Hutson P.R. (2020). The experimental effects of psilocybin on symptoms of anxiety and depression: A meta-analysis. Psychiatry Res..

[7] Romeo B., Karila L., Martelli C., Benyamina A. (2020). Efficacy of psychedelic treatments on depressive symptoms: A meta-analysis. J. Psychopharmacol..

[8] Carhart-Harris R.L., Roseman L., Bolstridge M., Demetriou L., Pannekoek J.N., Wall M.B., Tanner M., Kaelen M., McGonigle J., Murphy K. (2017). Psilocybin for treatment-resistant depression: fMRI-measured brain mechanisms. Sci. Rep..

[9] Mithoefer M.C., Grob C.S., Brewerton T.D. (2016). Novel psychopharmacological therapies for psychiatric disorders: Psilocybin and MDMA. Lancet Psychiatry.

[10] Benet L.Z., Kroetz D., Sheiner L., Hardman J., Limbird L. (1996). Pharmacokinetics: The dynamics of drug absorption, distribution, metabolism, and elimination. Goodman Gilman’s Pharmacol. Basis Ther..

[11] Ruiz-Garcia A., Bermejo M., Moss A., Casabo V.G. (2008). Pharmacokinetics in drug discovery. J. Pharm. Sci..

[12] Dinis-Oliveira R.J. (2017). Metabolism of psilocybin and psilocin: Clinical and forensic toxicological relevance. Drug Metab. Rev..

[13] Brown R.T., Nicholas C.R., Cozzi N.V., Gassman M.C., Cooper K.M., Muller D., Thomas C.D., Hetzel S.J., Henriquez K.M., Ribaudo A.S. (2017). Pharmacokinetics of escalating doses of oral psilocybin in healthy adults. Clin. Pharmacokinet..

[14] Papaseit E., Torrens M., Pérez-Mañá C., Muga R., Farré M. (2018). Key interindividual determinants in MDMA pharmacodynamics. Expert Opin. Drug Metab. Toxicol..

[15] Page M.J., McKenzie J.E., Bossuyt P.M., Boutron I., Hoffmann T.C., Mulrow C.D., Shamseer L., Tetzlaff J.M., Akl E.A., Brennan S.E. (2021). The PRISMA 2020 statement: An updated guideline for reporting systematic reviews. Int. J. Surg..

[16] Joanna Briggs Institute (2017). Checklist for Systematic Reviews and Research Syntheses. https://jbi.global/critical-appraisal-tools.

[17] Schneider K., Schwarz M., Burkholder I., Kopp-Schneider A., Edler L., Kinsner-Ovaskainen A., Hartung T., Hoffmann S. (2009). “ToxRTool”, a new tool to assess the reliability of toxicological data. Toxicol. Lett..

[18] Campbell M., McKenzie J.E., Sowden A., Katikireddi S.V., Brennan S.E., Ellis S., Hartmann-Boyce J., Ryan R., Shepperd S., Thomas J. (2020). Synthesis without meta-analysis (SWiM) in systematic reviews: Reporting guideline. BMJ.

[19] Becker A.M., Holze F., Grandinetti T., Klaiber A., Toedtli V.E., Kolaczynska K.E., Duthaler U., Varghese N., Eckert A., Grünblatt E. (2022). Acute Effects of Psilocybin After Escitalopram or Placebo Pretreatment in a Randomized, Double-Blind, Placebo-Controlled, Crossover Study in Healthy Subjects. Clin. Pharmacol. Ther..

[20] Chen J., Li M., Yan X., Wu E., Zhu H., Lee K.J., Chu V.M., Zhan L., Lee W., Kang J.S. (2011). Determining the pharmacokinetics of psilocin in rat plasma using ultra-performance liquid chromatography coupled with a photodiode array detector after orally administering an extract of Gymnopilus spectabilis. J. Chromatogr. B Anal. Technol. Biomed. Life Sci..

[21] Donovan L.L., Johansen J.V., Ros N.F., Jaberi E., Linnet K., Johansen S.S., Ozenne B., Issazadeh-Navikas S., Hansen H.D., Knudsen G.M. (2021). Effects of a single dose of psilocybin on behaviour, brain 5-HT2A receptor occupancy and gene expression in the pig. Eur. Neuropsychopharmacol..

[22] Hasler F., Bourquin D., Brenneisen R., Bär T., Vollenweider F.X. (1997). Determination of psilocin and 4-hydroxyindole-3-acetic acid in plasma by HPLC-ECD and pharmacokinetic profiles of oral and intravenous psilocybin in man. Pharm. Acta Helv..

[23] Hasler F., Bourquin D., Brenneisen R., Vollenweider F.X. (2002). Renal excretion profiles of psilocin following oral administration of psilocybin: A controlled study in man. J. Pharm. Biomed. Anal..

[24] Holze F., Ley L., Müller F., Becker A.M., Straumann I., Vizeli P., Kuehne S.S., Roder M.A., Duthaler U., Kolaczynska K.E. (2022). Direct comparison of the acute effects of lysergic acid diethylamide and psilocybin in a double-blind placebo-controlled study in healthy subjects. Neuropsychopharmacology.

[25] Horita A., Weber L.J. (1961). The enzymic dephosphorylation and oxidation of psilocybin and psilocin by mammalian tissue homogenates. Biochem. Pharmacol..

[26] Kolaczynska K.E., Liechti M.E., Duthaler U. (2021). Development and validation of an LC-MS/MS method for the bioanalysis of psilocybin’s main metabolites, psilocin and 4-hydroxyindole-3-acetic acid, in human plasma. J. Chromatogr. B Analyt. Technol. Biomed. Life Sci..

[27] Ley L., Holze F., Arikci D., Becker A.M., Straumann I., Klaiber A., Coviello F., Dierbach S., Thomann J., Duthaler U. (2023). Comparative acute effects of mescaline, lysergic acid diethylamide, and psilocybin in a randomized, double-blind, placebo-controlled cross-over study in healthy participants. Neuropsychopharmacology.

[28] Manevski N., Kurkela M., Höglund C., Mauriala T., Court M.H., Yli-Kauhaluoma J., Finel M. (2010). Glucuronidation of psilocin and 4-hydroxyindole by the human UDP-glucuronosyltransferases. Drug Metab. Dispos..

[29] Raithatha S.A., Hagel J.M., Matinkhoo K., Yu L., Press D., Cook S.G., Sharma G., Dhananjaya D., Jensen G., Lee J.B. (2024). Novel Psilocin Prodrugs with Altered Pharmacological Properties as Candidate Therapies for Treatment-Resistant Anxiety Disorders. J. Med. Chem..

[30] Rakoczy R.J., Runge G.N., Sen A.K., Sandoval O., Wells H.G., Nguyen Q., Roberts B.R., Sciortino J.H., Gibbons WJJr Friedberg L.M., Jones J.A. (2024). Pharmacological and behavioural effects of tryptamines present in psilocybin-containing mushrooms. Br. J. Pharmacol..

[31] Thomann J., Kolaczynska K.E., Stoeckmann O.V., Rudin D., Vizeli P., Hoener M.C., Pryce C.R., Vollenweider F.X., Liechti M.E., Duthaler U. (2024). In vitro and in vivo metabolism of psilocybin’s active metabolite psilocin. Front. Pharmacol..

[32] Richter L.M., Al-Gousous J., de Araujo G.L., Davies N.M., Löbenberg R. (2024). Assessing the utility of in silico tools in early drug development: The case of a pharmaceutically relevant formulation of the prodrug psilocybin. J. Drug Deliv. Sci. Technol..

[33] Sim D.S.M. (2015). Drug absorption and bioavailability. Pharmacological Basis of Acute Care.

[34] Stoner K.L., Harder H., Fallowfield L.J., Jenkins V.A. (2015). Intravenous versus subcutaneous drug administration. Which do patients prefer? A systematic review. Patient-Patient-Centered Outcomes Res..

[35] Dodd S., Norman T.R., Eyre H.A., Stahl S.M., Phillips A., Carvalho A.F., Berk M. (2023). Psilocybin in neuropsychiatry: A review of its pharmacology, safety, and efficacy. CNS Spectr..

[36] Roscoe J., Lozy O. (2022). Can psilocybin be safely administered under medical supervision? A systematic review of adverse event reporting in clinical trials. Drug Sci. Policy Law.

[37] Smith D.A., Beaumont K., Maurer T.S., Di L. (2015). Volume of distribution in drug design: Miniperspective. J. Med. Chem..

[38] Kroll R.A., Pagel M.A., Muldoon L.L., Roman-Goldstein S., Neuwelt E.A. (1996). Increasing volume of distribution to the brain with interstitial infusion: Dose, rather than convection, might be the most important factor. Neurosurgery.

[39] Otto M.E., van der Heijden K.V., Schoones J.W., van Esdonk M.J., Borghans L.G., Jacobs G.E., van Hasselt C.J. (2025). Clinical Pharmacokinetics of Psilocin After Psilocybin Administration: A Systematic Review and Post-Hoc Analysis. Clin. Pharmacokinet..

[40] Smith D.A., Beaumont K., Maurer T.S., Di L. (2017). Relevance of half-life in drug design: Miniperspective. J. Med. Chem..

[41] Meshkat S.J., Zeifman R., Stewart K., Janssen-Aguilar R., Lou W., Jetly R., Monson C.M., Bhat V. (2025). Psilocybin-assisted massed cognitive processing therapy for chronic posttraumatic stress disorder: Protocol for an open-label pilot feasibility trial. PLoS ONE.

[42] Husain M.I., Blumberger D.M., Castle D.J., Ledwos N., Fellows E., Jones B.D., Ortiz A., Kloiber S., Wang W., Rosenblat J.D. (2023). Psilocybin for treatment-resistant depression without psychedelic effects: Study protocol for a 4-week, double-blind, proof-of-concept randomised controlled trial. BJPsych Open.

[43] Deodhar M., Al Rihani S.B., Arwood M.J., Darakjian L., Dow P., Turgeon J., Michaud V. (2020). Mechanisms of CYP450 inhibition: Understanding drug-drug interactions due to mechanism-based inhibition in clinical practice. Pharmaceutics.

[44] Lynch T., Price A.M. (2007). The effect of cytochrome P450 metabolism on drug response, interactions, and adverse effects. Am. Fam. Physician.

[45] Halman A., Kong G., Sarris J., Perkins D. (2023). Drug-drug interactions between classic psychedelics and psychoactive drugs: A systematic review. medRxiv.

[46] Boyd-Kimball D., Gonczy K., Lewis B., Mason T., Siliko N., Wolfe J. (2018). Classics in chemical neuroscience: Chlorpromazine. ACS Chem. Neurosci..

[47] Vollenweider F.X., Vollenweider-Scherpenhuyzen M.F., Bäbler A., Vogel H., Hell D. (1998). Psilocybin induces schizophrenia-like psychosis in humans via a serotonin-2 agonist action. Neuroreport.

[48] (1958). LESSES Psychodynamic relationships between the degree of anxiety and other clinical symptoms. J. Nerv. Ment. Dis..

[49] Erkizia-Santamaría I., Alles-Pascual R., Horrillo I., Meana J.J., Ortega J.E. (2022). Serotonin 5-HT2A, 5-HT2c and 5-HT1A receptor involvement in the acute effects of psilocybin in mice. In vitro pharmacological profile and modulation of thermoregulation and head-twich response. Biomed. Pharmacother..

[50] Goodwin G.M., Croal M., Feifel D., Kelly J.R., Marwood L., Mistry S., O’Keane V., Peck S.K., Simmons H., Sisa C. (2023). Psilocybin for treatment resistant depression in patients taking a concomitant SSRI medication. Neuropsychopharmacology.

[51] Simonsson O., Hendricks P.S., Chambers R., Osika W., Goldberg S.B. (2023). Prevalence and associations of challenging, difficult or distressing experiences using classic psychedelics. J. Affect. Disord..

[52] Nayak S.M., Gukasyan N., Barrett F.S., Erowid E., Erowid F., Griffiths R.R. (2021). Classic psychedelic coadministration with lithium, but not lamotrigine, is associated with seizures: An analysis of online psychedelic experience reports. Pharmacopsychiatry.

[53] Ali A., Gifford M.E., Lowe H., Gordon L., Grant J. (2023). Natural vs. Synthetic Psilocybin: The Same or Completely Different?. Mushrooms with Therapeutic Potentials: Recent Advances in Research and Development.

[54] Lowe H., Toyang N., Steele B., Valentine H., Grant J., Ali A., Ngwa W., Gordon L. (2021). The therapeutic potential of psilocybin. Molecules.

[55] Johnson M.W., Griffiths R.R. (2017). Potential therapeutic effects of psilocybin. Neurotherapeutics.

[56] Smigielski L., Scheidegger M., Kometer M., Vollenweider F.X. (2019). Psilocybin-assisted mindfulness training modulates self-consciousness and brain default mode network connectivity with lasting effects. NeuroImage.

[57] Gattuso J.J., Perkins D., Ruffell S., Lawrence A.J., Hoyer D., Jacobson L.H., Timmermann C., Castle D., Rossell S.L., Downey L.A. (2023). Default mode network modulation by psychedelics: A systematic review. Int. J. Neuropsychopharmacol..

[58] Zhao X., Du Y., Yao Y., Dai W., Yin Y., Wang G., Li Y., Zhang L. (2024). Psilocybin promotes neuroplasticity and induces rapid and sustained antidepressant-like effects in mice. J. Psychopharmacol..

[59] Rosenblat J.D., Meshkat S., Doyle Z., Kaczmarek E., Brudner R.M., Kratiuk K., Mansur R.B., Schulz-Quach C., Sethi R., Abate A. (2024). Psilocybin-assisted psychotherapy for treatment resistant depression: A randomized clinical trial evaluating repeated doses of psilocybin. Med.

[60] Haikazian S., Chen-Li D.C., Johnson D.E., Fancy F., Levinta A., Husain M.I., Mansur R.B., McIntyre R.S., Rosenblat J.D. (2023). Psilocybin-assisted therapy for depression: A systematic review and meta-analysis. Psychiatry Res..

