# Supplementary material for: Pharmacokinetics of Psilocybin: A Systematic Review
Source: Pharmaceutics. 2025 Mar 25;17(4):411. doi: 10.3390/pharmaceutics17040411 (PMC12030428; doi:10.3390/pharmaceutics17040411)
Supplement: Supplementary file 1 [file pharmaceutics-17-00411-s001.zip › pharmaceutics-3522658-supplementary.pdf]

Supplementary materials

Table S1. Full search strategy.

1. Psilocybin/
2. (psilocyb* or psilocib* or psilocin* or silocyb* or shrooms or magic mushrooms or mushies or psilocybin-assisted therapy).tw, kf.
3. 1 or 2
4. exp Pharmacokinetics/
5. Metabolism/
6. exp Biotransformation/
7. exp cytochromes/
8. Enzymes/
9. (metaboli* or pharmacokinetic* or biotransformation or Cytochrome P450 or CYP450 or CYP enzymes or CYP2D6 or CYP1A2 or CYP3A4 or drug-drug interaction or therapeutic* or safe* or drug interaction* or side effect*).tw,kf.
10. (Enzyme adj5 (activit* or inhibit*)).tw,kf.
11. 4 or 5 or 6 or 7 or 8 or 9 or 10
12. (psyc* or mental health or mental disorder* or borderline personality disorder or BPD or trauma* or PTSD or PTSI or postraumatic* or depressi* or obsessive compulsive disorder* or OCD or bipolar or anxiety or mood* or depress* or MDD or SUD).tw,kf.
13. 3 and 11 and 12

Table S2. Risk of bias assessment results.

Quality Assessment using JBI Tools for Included Articles

Randomized Controlled Trial															
Author	Year	Q1: Was true randomization used for assignment of participants to treatment groups?	Q2: Was allocation to treatment groups concealed?	Q3: Were treatment groups similar at the baseline?	Q4: Were participants blind to treatment assignment?	Q5: Were those delivering the treatment blind to treatment assignment?	Q6: Were treatment groups treated identically other than the intervention of interest?	Q7: Were outcome assessors blind to treatment assignment?	Q8: Were outcomes measured in the same way for treatment groups?	Q9: Were outcomes measured in a reliable way? (Each outcome)	Q10: Was follow-up complete and, if not, were differences between groups in terms of their follow-up adequately described and analyzed?	Q11: Were participants analyzed in the groups to which they were randomized?	Q12: Was appropriate statistical analysis used?	Q13: Was the trial design appropriate and any deviations from the standard RCT design (individual randomization, parallel groups) accounted for in the conduct and analysis of the trial?	Overall Appraisal
Becker et al.	2022	Yes	Yes	Yes	Yes	Yes	Yes	Yes	Yes	Yes	No	Yes	Yes	Yes	Include
Holze et al.	2022	Yes	Yes	Yes	Yes	Yes	Yes	Yes	Yes	Yes	No	Yes	Yes	Yes	Include
Ley et al.	2023	Yes	Yes	Yes	Yes	Yes	Yes	Yes	Yes	Yes	Yes	Yes	Yes	Yes	Include

Quasi-Experimental Studies											
Author	Year	Q1: Is it clear in the study what is the "cause"	Q2: Was there a	Q3: Were participants	Q4: Were the participants included in any compari-	Q5: Were there multiple measurements of	Q6: Were the outcomes of partici-	Q7: Were outcomes measured in a reliable	Q8: Was follow-up complete and if not, were differences between	Q9: Was appropriate statistical analysis used?	Overall Appraisal

		and what is the “effect” (i.e. there is no confusion about which variable comes first)?	control group?	included in any comparisons similar?	sons receiving similar treatment/care, other than the exposure or intervention of interest?	the outcome, both pre and post the intervention/exposure? (Each outcome)	pants included in any comparisons measured in the same way? (Each outcome)	way? (Each outcome)	groups in terms of their follow-up adequately described and analyzed? (Each outcome)	(Each outcome)	
Brown et al.	2017	Yes	No	Yes	Yes	Yes	Yes	Yes	Yes	Yes	Include
Hasler et al.	1997	Yes	No	Yes	Yes	Yes	Yes	Yes	Unclear	Yes	Include
Hasler et al.	2022	Yes	No	Yes	Yes	Yes	Yes	Yes	Yes	Yes	Include
Holze et al.	2022	Yes	No	Yes	Yes	Yes	Yes	Yes	No	Yes	Include
Nicholas et al.	2017	Yes	No	Yes	Yes	Yes	Yes	Yes	Yes	Yes	Include

Quality Assessment Results for Included In Vivo Toxicity Studies

Authors	Year	Criteria Group I: Test substance identification	Criteria Group II: Test organism characterisation	Criteria Group III: Study design description	Criteria Group IV: Study results documentation	Criteria Group V: Plausibility of study design and results	A Numerical result leads to initial Category:	B Checking red scores leads to revised Category:	C Evaluator's proposal: Category:	D Justification in case evaluator deviates from B:
Chen et al.	2011	4/4	4/5	7/7	3/3	2/2	1	1	1	NA
Donovan et al.	2021	4/4	5/5	7/7	3/3	2/2	1	1	1	NA

Kolaczyn-ska et al.	2021	4/4	3/5	7/7	3/3	2/2	1	1	1	NA
Thomann et al.	2024	4/4	3/5	7/7	3/3	2/2	1	1	1	NA
Rakoczy et al.	2024	4/4	4/5	7/7	3/3	2/2	1	1	1	NA
Raithatha et al.	2023	4/4	5/5	7/7	3/3	2/2	1	1	1	NA

Quality Assessment Results for Included In Vitro Toxicity Studies

Authors	Year	Criteria Group I: Test substance identification	Criteria Group II: Test system characterisation	Criteria Group III: Study design description	Criteria Group IV: Study results documentation	Criteria Group V: Plausibility of study design and data	A Numerical result leads to initial Category:	B Checking red scores leads to revised Category:	C Evaluator's proposal: Category:	D Justification in case evaluator deviates from B:
Horita & Weber	1961	4/4	3/3	6/6	3/3	2/2	1	1	1	NA
Manevski et al.	2010	4/4	2/3	5/6	3/3	2/2	1	1	1	NA
Raithatha et al.	2024	4/4	3/3	6/6	3/3	2/2	1	1	1	NA
Thomann et al.	2024	4/4	2/3	6/6	3/3	2/2	1	1	1	NA
Rakoczy et al.	2024	4/4	3/3	6/6	3/3	2/2	1	1	1	NA

Table S3 PRISMA Checklists

Section and Topic	Item #	Checklist item	Location where item is reported
**TITLE**			
Title	1	Identify the report as a systematic review.	1
**ABSTRACT**			
Abstract	2	See the PRISMA 2020 for Abstracts checklist.	2
**INTRODUCTION**			
Rationale	3	Describe the rationale for the review in the context of existing knowledge.	3,4
Objectives	4	Provide an explicit statement of the objective(s) or question(s) the review addresses.	4
**METHODS**			
Eligibility criteria	5	Specify the inclusion and exclusion criteria for the review and how studies were grouped for the syntheses.	5
Information sources	6	Specify all databases, registers, websites, organisations, reference lists and other sources searched or consulted to identify studies. Specify the date when each source was last searched or consulted.	5
Search strategy	7	Present the full search strategies for all databases, registers and websites, including any filters and limits used.	5
Selection process	8	Specify the methods used to decide whether a study met the inclusion criteria of the review, including how many reviewers screened each record and each report retrieved, whether they worked independently, and if applicable, details of automation tools used in the process.	5
Data collection process	9	Specify the methods used to collect data from reports, including how many reviewers collected data from each report, whether they worked independently, any processes for obtaining or confirming data from study investigators, and if applicable, details of automation tools used in the process.	5,6
Data items	10a	List and define all outcomes for which data were sought. Specify whether all results that were compatible with each outcome domain in each study were sought (e.g. for all measures, time points, analyses), and if not, the methods used to decide which results to collect.	5,6
	10b	List and define all other variables for which data were sought (e.g. participant and intervention characteristics, funding sources). Describe any assumptions made about any missing or unclear information.	5,6
Study risk of bias assessment	11	Specify the methods used to assess risk of bias in the included studies, including details of the tool(s) used, how many reviewers assessed each study and whether they worked independently, and if applicable, details of automation tools used in the process.	6
Effect measures	12	Specify for each outcome the effect measure(s) (e.g. risk ratio, mean difference) used in the synthesis or presentation of results.	NA
Synthesis methods	13a	Describe the processes used to decide which studies were eligible for each synthesis (e.g. tabulating the study intervention characteristics and comparing against the planned groups for each synthesis (item #5)).	7
	13b	Describe any methods required to prepare the data for presentation or synthesis, such as handling of missing summary statistics, or data conversions.	NA

Section and Topic	Item #	Checklist item	Location where item is reported
	13c	Describe any methods used to tabulate or visually display results of individual studies and syntheses.	7
	13d	Describe any methods used to synthesize results and provide a rationale for the choice(s). If meta-analysis was performed, describe the model(s), method(s) to identify the presence and extent of statistical heterogeneity, and software package(s) used.	7
	13e	Describe any methods used to explore possible causes of heterogeneity among study results (e.g. subgroup analysis, meta-regression).	NA
	13f	Describe any sensitivity analyses conducted to assess robustness of the synthesized results.	NA
Reporting bias assessment	14	Describe any methods used to assess risk of bias due to missing results in a synthesis (arising from reporting biases).	NA
Certainty assessment	15	Describe any methods used to assess certainty (or confidence) in the body of evidence for an outcome.	NA
**RESULTS**			
Study selection	16a	Describe the results of the search and selection process, from the number of records identified in the search to the number of studies included in the review, ideally using a flow diagram.	7, Fig 1
	16b	Cite studies that might appear to meet the inclusion criteria, but which were excluded, and explain why they were excluded.	7
Study characteristics	17	Cite each included study and present its characteristics.	Table 1
Risk of bias in studies	18	Present assessments of risk of bias for each included study.	Supplementary materials
Results of individual studies	19	For all outcomes, present, for each study: (a) summary statistics for each group (where appropriate) and (b) an effect estimate and its precision (e.g. confidence/credible interval), ideally using structured tables or plots.	7-21
Results of syntheses	20a	For each synthesis, briefly summarise the characteristics and risk of bias among contributing studies.	7-21
	20b	Present results of all statistical syntheses conducted. If meta-analysis was done, present for each the summary estimate and its precision (e.g. confidence/credible interval) and measures of statistical heterogeneity. If comparing groups, describe the direction of the effect.	NA
	20c	Present results of all investigations of possible causes of heterogeneity among study results.	NA
	20d	Present results of all sensitivity analyses conducted to assess the robustness of the synthesized results.	NA
Reporting biases	21	Present assessments of risk of bias due to missing results (arising from reporting biases) for each synthesis assessed.	NA
Certainty of evidence	22	Present assessments of certainty (or confidence) in the body of evidence for each outcome assessed.	NA
**DISCUSSION**			
Discussion	23a	Provide a general interpretation of the results in the context of other evidence.	21,22

Section and Topic	Item #	Checklist item	Location where item is reported
	23b	Discuss any limitations of the evidence included in the review.	26
	23c	Discuss any limitations of the review processes used.	26
	23d	Discuss implications of the results for practice, policy, and future research.	27
**OTHER INFORMATION**			
Registration and protocol	24a	Provide registration information for the review, including register name and registration number, or state that the review was not registered.	4
	24b	Indicate where the review protocol can be accessed, or state that a protocol was not prepared.	4
	24c	Describe and explain any amendments to information provided at registration or in the protocol.	NA
Support	25	Describe sources of financial or non-financial support for the review, and the role of the funders or sponsors in the review.	28
Competing interests	26	Declare any competing interests of review authors.	28,29
Availability of data, code and other materials	27	Report which of the following are publicly available and where they can be found: template data collection forms; data extracted from included studies; data used for all analyses; analytic code; any other materials used in the review.	NA

From: Page MJ, McKenzie JE, Bossuyt PM, Boutron I, Hoffmann TC, Mulrow CD, et al. The PRISMA 2020 statement: an updated guideline for reporting systematic reviews. BMJ 2021;372:n71. doi: 10.1136/bmj.n71.
This work is licensed under CC BY 4.0. To view a copy of this license, visit https://creativecommons.org/licenses/by/4.0/
